# Emergence and Clonal Spread of Extended-Spectrum β-Lactamase-Producing *Salmonella* Infantis Carrying pESI Megaplasmids in Korean Retail Poultry Meat

**DOI:** 10.3390/antibiotics14040366

**Published:** 2025-04-01

**Authors:** Yeona Kim, Hyeonwoo Cho, Miru Lee, Amany Hassan, Soo-Jin Yang, Jong-Chan Chae, Kun Taek Park

**Affiliations:** 1 Department of Digital Anti-Aging and Healthcare, Inje University, Gimhae 50834, Republic of Korea; april_k0526@naver.com; 2 Department of Biological Sciences, Inje University, Gimhae 50834, Republic of Korea; hu23749908@gmail.com (H.C.); leemiru456@gmail.com (M.L.); 3 Department of Veterinary Microbiology and Pathology, Washington State University, Pullman, WA 99164, USA; amany.hassan@wsu.edu; 4 Department of Animal Medicine, Faculty of Veterinary Medicine, Alexandria University, Alexandria 21944, Egypt; 5 Department of Veterinary Microbiology, College of Veterinary Medicine and Research Institute for Veterinary Science, Seoul National University, Seoul 08826, Republic of Korea; soojinjj@snu.ac.kr; 6 Division of Biotechnology, Jeonbuk National University, Iksan 54596, Republic of Korea; chae@jbnu.ac.kr

**Keywords:** poultry meat, extended-spectrum β-lactamase, *Salmonella* Infantis, *bla_CTX-M-65_*, pESI

## Abstract

**Background/Objectives:** *Salmonella* is a major cause of foodborne illnesses, with multidrug-resistant (MDR) strains posing significant threats to public health worldwide. This study investigated the prevalence and antimicrobial resistance (AMR) of *Salmonella*, focusing on extended-spectrum β-lactamase (ESBL)-producing *Salmonella* in retail poultry meat in Korea. **Methods**: A total of 300 poultry meat samples were collected nationwide from retail markets. Multi-locus sequence typing, serotyping, and antimicrobial susceptibility testing were performed. Whole-genome sequencing (WGS) analysis was conducted against 28 representative ESBL-producing *S*. Infantis isolates to identify the genetic characteristics and phylogenetic relationship. **Results**: *Salmonella* was detected in 81.3% of raw poultry meat samples, with *S*. Infantis ST32 being the dominant serotype in chicken (53.0%) and *S*. Typhimurium ST19 predominant in duck (39.0%). MDR was identified in 58.2% of samples, with a significantly higher rate in chicken isolates than in duck isolates (*p* < 0.001). Notably, 75.3% of chicken MDR isolates were ESBL-producing *S*. Infantis carrying *bla_CTX-M-65_*. WGS of 28 geographically and phenotypically representative ESBL-producing *S*. Infantis revealed five clonal clusters, suggesting the widespread dissemination of ESBL-producing *S*. Infantis across Korea’s poultry supply chain. All 28 ESBL-producing *S*. Infantis isolates contained a pESI-like megaplasmid, carrying multiple resistance and virulence genes, with sequences highly identical to plasmids reported in the United States, indicating potential international transmission. **Conclusions**: This study emphasizes the urgent need for continuous surveillance and responsible antibiotic use in livestock under a One Health framework. WGS can provide an effective tool for tracking AMR evolution and clonal spread within and across regions.

## 1. Introduction

*Salmonella* is a major zoonotic foodborne pathogen with significant public health and economic impacts worldwide [1]. It causes more than 90 million cases of gastroenteritis, with an estimated annual salmonellosis incidence ranging from 200 million to more than 1 billion [2]. According to the Ministry of Food and Drug Safety (MFDS, Korea), *Salmonella* was responsible for 10,888 reported infections between 2013 and 2023, making it the third most common cause of foodborne illness in Korea after pathogenic *Escherichia coli* (15,715 cases) and norovirus (10,947 cases) [3]. *Salmonella* is typically found as a normal component of animal gut microbiota and serves as a source of transmission from animals to humans. Approximately 85% of *Salmonella*-associated foodborne illnesses are associated with the consumption of contaminated foods, including meat, eggs, and dairy products [4,5]. To date, approximately 2600 *Salmonella* serovars have been identified and classified as typhoidal or non-typhoidal based on their pathogenicity in humans and animals [4]. Non-typhoidal *Salmonella* (NTS) is associated with various serotypes and is a leading cause of foodborne diarrhea [6]. Among the NTS serotypes, *S*. Typhimurium and *S*. Enteritidis are the most common causes of zoonotic infection of humans and are usually associated with poultry and poultry products [7]. The most prevalent serotypes related to human infections include Enteritidis, Newport, Typhimurium, Javiana, and monophasic Typhimurium 4,[5],12:i:- in the United States and Enteritidis, Typhimurium, monophasic Typhimurium, Infantis, and Newport in the European Union [7].

Antimicrobials have been widely used for prophylaxis and treatment in livestock industries for several decades [8,9]. Moreover, antimicrobials have also been used at subtherapeutic doses to promote growth by regulating intestinal microbiota [10]. However, the emergence of bacteria with antimicrobial resistance (AMR) has led several countries to ban their use for growth promoters [10,11]. Despite these measures, the prolonged and often inappropriate use of antimicrobials places selective pressure on bacteria in animals and the environment, facilitating resistance [12]. The emergence of multidrug-resistant (MDR) *Salmonella* strains has gradually increased, leading to significant health concerns owing to the potential for treatment failure of salmonellosis [9,13]. Resistance to extended-spectrum β-lactams, particularly third- and fourth-generation cephalosporins and carbapenems, poses a significant threat, as they are critically important antimicrobials in human and veterinary medicine [9].

A particularly concerning development is the global emergence of MDR-emergent *S*. Infantis (ESI), representing a challenge for the poultry industry [14]. These strains carry a large plasmid, known as the *pESI* or pESI-like plasmid, which encodes multiple AMR genes, including the extended-spectrum β-lactamase (ESBL) gene [15]. The rising prevalence of ESI in poultry farms and meat products has led to a rise in *S*. Infantis infections in the United States, South America, and Europe, primarily linked to contaminated chicken products, posing serious risks to both animals and humans [14,16,17,18].

In Korea, the emergence of ESBL-producing MDR *S*. Infantis was first reported in chicken and duck samples collected from slaughterhouses in 2020 and 2021, respectively [19]. Although not characterized in detail, these isolates were presumed to be ESI strains carrying pESI or pESI-like plasmids. Recently, the presence of ESI carrying pESI-like plasmids was confirmed in broiler farms and egg grading and packing plants through PCR-based detection [20,21]. However, the molecular characterization of these ESI strains was limited due to the methodologies used, such as pulsed-field gel electrophoresis (PFGE) and multiplex PCRs, which provide limited genetic information and make cross-comparisons with international ESI strains challenging. Therefore, we investigated the nationwide distribution of ESBL-producing *Salmonella* spp., as well as ESI carrying pESI-like plasmids, in retail chicken and duck meat in South Korea. We employed the advanced technique of whole-genome sequencing (WGS) to thoroughly characterize the genetic elements and clonal relationships of ESI isolates and to trace the origins of pESI-like plasmids based on sequence identities.

## 2. Results

### 2.1. Prevalence, Serotype, and Sequence Type Distribution of Salmonella in Poultry Meats

The overall prevalence, serotypes, and sequence types (STs) of *Salmonella* spp. in 300 retail poultry meats are shown in Table 1 and Supplementary Table S1. *Salmonella* contamination was found in 81.3% (244 out of 300 samples) of retail poultry meat samples. There was no statistically significant difference between the prevalence of *Salmonella* in chicken (79.0%, 158 out of 200 samples) and duck (86.0%, 86 out of 100 samples) meat. Serotyping of the 244 isolates identified 11 serotypes, with an additional 34 isolates being non-typable. These isolates were associated with 18 STs, with most serotypes belonging to distinct STs. The distribution of serotypes and STs in chickens and ducks showed clear differences. The most prevalent serotype was *S*. Infantis (53.0%), followed by *S*. Agona (10.5%) in chicken, *S*. Typhimurium (39.0%), and *S*. Infantis and *S*. Brandenburg (7.0%) in duck meat. All the *S*. Infantis isolates belonged to ST32, whereas *S*. Typhimurium was classified as ST19. Additionally, significant differences were observed in the isolation rates of the eight STs between chicken and duck meats (*p* < 0.05).

Notably, based on the Korean Animal Product Traceability system, we found that all *S*. Infantis-positive duck meat was processed in slaughterhouses processing both chickens and ducks, whereas all duck meat processed in slaughterhouses that only handle ducks was negative for *S*. Infantis.

### 2.2. Comparisons of AMR Profiles of Salmonella enterica Isolates from Chicken and Duck Meat

Overall, 93.7% (n = 148) of *Salmonella* isolates from chicken meat samples were resistant to at least one antimicrobial agent, and 79.7% (n = 126) of the isolates exhibited an MDR phenotype. In contrast, 58.1% (n = 50) of duck meat isolates were resistant to at least one antimicrobial agent, and 18.6% (n = 16) were MDR (Table 2). Among 244 *Salmonella* isolates, the highest resistance was observed against NAL (68.0%), followed by TET (54.9%), STR, and FIS (54.5%). Chicken isolates exhibited significantly higher resistance to AMP, CHL, CTX, FIS, GEN, NAL, STR, STX, and TET than that of duck isolates (*p* < 0.001). Moreover, resistance to AUG2, FEP, FOX, GEN, and CAZ was detected only in the chicken meat isolates. Notably, all the 244 isolates were susceptible to MERO.

### 2.3. ESBL-Producing Salmonella

According to the results of the antimicrobial susceptibility test, 63.3% (n = 100) and 8.1% (n = 7) of chicken and duck meat isolates, respectively, were ESBL-producing *Salmonella*, which showed resistance against CTX (≥16 µg/mL). Interestingly, all ESBL-producing *Salmonella* isolates were *S*. Infantis (Table 2). PCR screening of ESBL-encoding genes revealed that all ESBL-producing *S*. Infantis harbored the *bla_CTX-M-65_* gene, belonging to the CTX-M-9 group. All ESBL-producing isolates tested negative for *bla_TEM_* and *bla_SHV_*, besides one isolate from chicken meat that harbored *bla_TEM-98_*. All ESBL-producing isolates were MDR and resistant to at least five classes of antimicrobials (Table 3). Most ESBL-producing isolates were resistant to CTX, NAL, AMP (all 100.0%), CHL (99.1%), TET (95.3%), and FIS (92.5%). Twenty-two combinations of AMR patterns were identified. The most common combination of AMR patterns was resistance to AMP-CHL-CTX-FIS-STR-SXT-NAL-TET (41.1%), followed by resistance to AMP-CHL-CTX-FIS-GEN-STR-SXT-NAL-TET and AMP-CHL-CTX-FIS-NAL-STR-TET (15.9%).

### 2.4. Genomic Characteristics of ESBL-Producing S. Infantis

To further investigate the genetic characteristics and clonal relationships of ESBL-producing *Salmonella*, representative ESBL-producing *S*. Infantis isolates were selected for WGS. For chicken isolates, the three most common AMR patterns (Table 3) were detected across all five provinces. The AMP-CHL-CTX-NAL-TET resistance pattern was detected in three provinces (Gyeonggi, Chungcheong, and Gangwon provinces), while AMP-CHL-CTX-FIS-GEN-NAL-STR-TET resistance was detected in two provinces (Gyeonggi and Chungcheong provinces). One isolate with each AMR pattern was selected from each province, resulting in the selection of twenty isolates. The two isolates that showed resistance to the highest numbers of antimicrobial agents (12 and 11 antimicrobials, Table 3) were included. Three isolates from different retail markets were additionally selected to represent all ESBL-producing *S*. Infantis-positive retail markets (19 retail markets).

For duck isolates, ESBL-producing *S*. Infantis was only detected in three markets, all located in Chungcheong Province; all of them showed the same AMR pattern (Table 3). One from each market was included in the WGS analysis. Taken together, 25 chicken-derived and 3 duck-derived ESBL-producing *S*. Infantis isolates were subjected to WGS.

**Figure 1:**
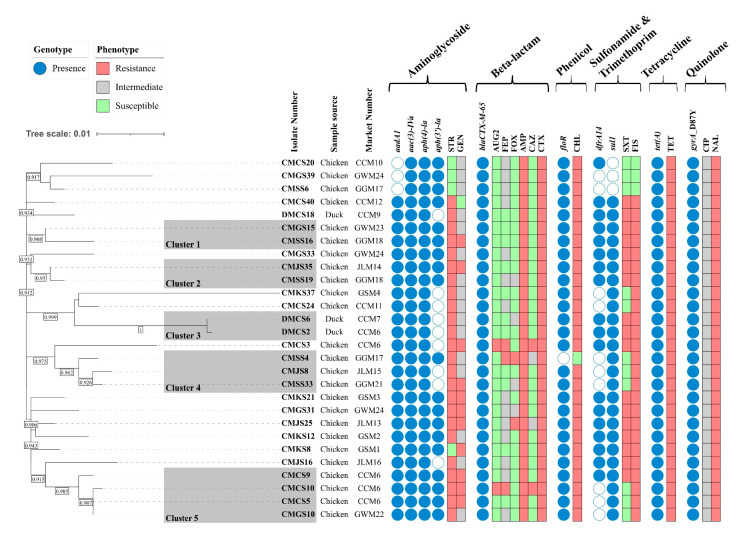
Phylogenetic tree and AMR profiles of 28 ESBL-producing *S.* Infantis samples. A maximum-likelihood phylogenetic tree of 28 ESBL-producing *S*. Infantis samples was constructed using the FDA-CFSAN SNP pipeline and FastTree using *S*. Infantis SINFA (N649235) as a reference strain. Genetically related isolates were clustered by the framework provided by the FDA. Letters beside the isolate number indicate the sample sources. The bootstraps higher than 0.90 are shown in the box between the separated tree nodes. The presence and absence of AMR genes are indicated in blue and white circles, respectively. The AMR profiles are indicated in red (resistance), gray (intermediate), and green (susceptible) boxes, respectively. Market number: GSM, Gyeongsang Market; CCM, Chungcheong Market; JLM, Jeolla Market; GGM, Gyeonggi Market; GWM, Gangwon Market. AMP, ampicillin; AUG2, amoxicillin/clavulanic acid; CAZ, ceftazidime; CHL, chloramphenicol; CIP, ciprofloxacin; CTX, cefotaxime; FEP, cefepime; FIS, sulfisoxazole; FOX, cefoxitin; GEN, gentamicin; NAL, nalidixic acid; STR, streptomycin; SXT, trimethoprim/sulfamethoxazole; TET, tetracycline.

The in silico serotyping revealed that all 28 ESBL-producing isolates were *Salmonella enterica* serovar Infantis (antigenic formula: 7:r:1,5). To assess the genetic relatedness among the 28 ESBL-producing isolates, an ML phylogenetic tree was constructed based on 1422 SNPs in the core gene alignment. Five clusters were generated, and most isolates clustered together in Clusters 1–5, regardless of their geographical region. The isolates in Cluster 1 (10 SNP differences), Cluster 2 (3 SNP differences), Cluster 4 (median 8.7, range 6–11 SNP differences), and Cluster 5 (median 4, range 0–8 SNP differences) were clustered together, even though they were collected from different geographical regions. Moreover, two isolates collected from the Chungcheong (CMCS5) and Gangwon (CMGS10) provinces in Cluster 5 were identical, with no SNP differences. Among the three duck isolates, DMCS18 belonged to a singleton, but the remaining two isolates (DMCS2 and DMCS6) clustered together in Cluster 3, with only one SNP difference. These two isolates were collected from duck meat purchased from different markets but processed in a slaughterhouse that processed chicken and ducks together, suggesting possible cross-contamination between chicken and duck meat during slaughter. Taken together, the average SNP differences among the isolates within the same clusters were ≤10, indicating close genetic relatedness among the *S*. Infantis isolates (Figure 1).

The AMR genotypes and phenotypes of the 28 ESBL-producing *S*. Infantis isolates are shown in Figure 1. A total of nine AMR genes and one point mutation were detected in this study. Genes conferring resistance to aminoglycoside (*aadA1*, *aph(4)-Ia*, *aac(3)-IVa*, *aph(3′)-Ia*), β-lactam (*bla_CTX-M-65_*), phenicol, (*floR*), quinolone (gyrA_D87Y point mutation), sulfonamide (*sul1*), trimethoprim (*dfrA14*), and tetracycline (*tet(A)*) were identified. Plasmid-mediated quinolone resistance (PMQR) genes were not detected in this study. All 28 isolates harbored *aac(3)-IVa*, *aph(4)-Ia*, *tet(A)*, *bla_CTX-M-65_*, and D87Y point mutations in *gyrA* (100% prevalence). Most of the AMR genotypes of the ESBL-producing *Salmonella* isolates corresponded to AMR phenotypes.

### 2.5. Characterization of pESI-like Plasmids

A plasmid replicon search revealed that only one type of plasmid replicon, IncFIB (pN55391), was present in all 28 *S*. Infantis isolates.

Plasmids were reconstructed using the mob-suite tool to further analyze the characteristics of the pESI-like megaplasmid in ESBL-producing *S*. Infantis isolates. All 28 ESBL-producing *S*. Infantis isolates carried a conjugative plasmid with predicted sizes ranging from 300,523 bp to 312,318 bp (Supplementary Table S2). IncI1 pMLST of the plasmids was positive for four genes (*ardA*, *pilL*, *sogS*, *trbA*), whereas all isolates were negative for the replicase gene *repl1*. Among the plasmid allele profiles, 26 of the 28 isolates displayed an allele combination of *ardA*_11, *pilL*_3, *sogS*_14, and *trbA*_8. Two plasmids (pCMKS37 and pCMGS31) showed a one-allele difference in *ardA* (*ardA*_3). Genes conferring resistance to aminoglycoside (*aadA1*, *aph(4)-Ia*, *aac(3)-IVa*, *aph(3′)-Ia*), sulfonamide (*sul1*), tetracycline (*tet(A)*), phenicol (*floR*), trimethoprim (*dfrA14*), and β-lactam (*bla_CTX-M-65_*) were detected in the plasmids, except for one isolate (CMCS20), in which *dfrA14* was found in the chromosome. Virulence genes associated with yersiniabactin biosynthesis (*irp1*, *irp2*, *fyuA*/*psn*, *fyuA*, *ybtA*, *ybtE*, *ybtP*, *ybtQ*, *ybtS*, *ybtT*, *ybtU*, *ybtX*), along with genes conferring resistance to mercury (*merC*, *merP*, *merT*, *merR*) and quaternary ammonium compounds (*qacEΔ1*), which are frequently identified in pESI-like plasmids, were detected in all 28 plasmids.

The mob-typer results revealed that all plasmids identified in this study were identical to the pESI plasmid carrying *bla_CTX-M-65_*, specifically pCVM44454 (GenBank accession no. CP016413.1) and pN55391 (CP016411.1), which were previously reported in the United States.

To place these findings in a global context, we compared the plasmid sequences from our isolates with representative pESI-like plasmids reported from other countries, including Israel (119944_pESI), Italy (12037823-11_Italy_pESI_CTX-M-1), and Slovenia (pS19). The pESI-like plasmids in this study showed the highest average nucleotide identity (ANI) values when compared to those from the United States (99.99–100%). In contrast, ANI values for the plasmids from Israel, Italy, and Slovenia ranged from 99.12% to 99.64% (Supplementary Table S3).

**Figure 2:**
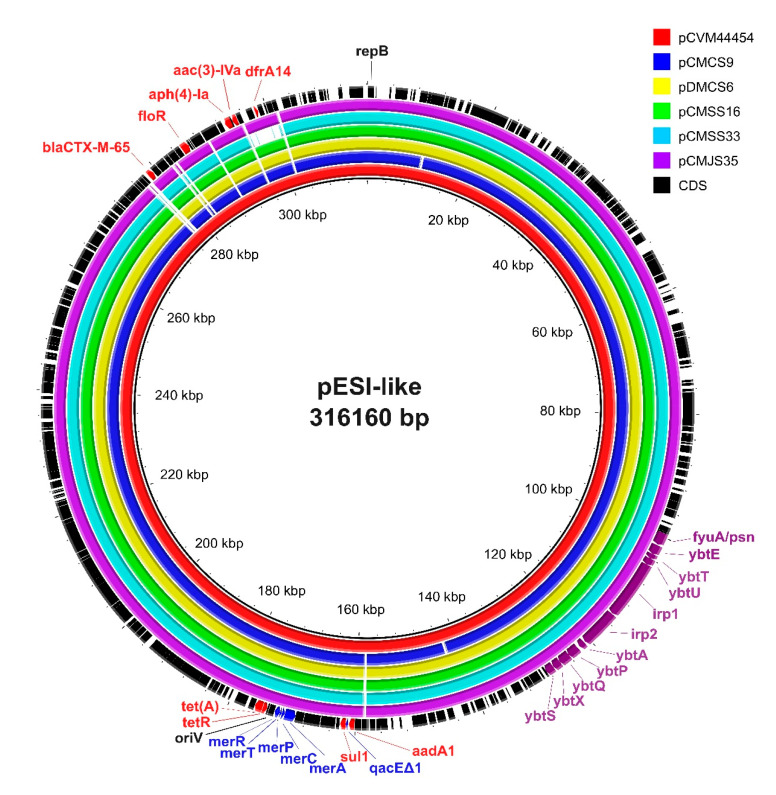
Comparative genomic analysis of emerging *Salmonella enterica* Infantis (pESI)-like plasmids. Sequence alignment of pESI-like plasmids in extended-spectrum β-lactamase (ESBL)-producing *S*. Infantis was performed using BRIG software (v.0.95). The plasmid pCVM44454 (CP016413.1) was used as the reference plasmid. The respective plasmids corresponded with the colors shown in the legend on the right. Structurally similar regions are shown with colors, whereas the missing regions are shown as blank. Antimicrobial resistance-, stress resistance-, virulence-, and replication-related genes are indicated in red, blue, purple, and black letters, respectively.

For further structural comparison, one isolate from each of the five phylogenetic clusters was selected and aligned with pCVM44454. All five representative plasmids exhibited nearly identical structures to pCVM44454, except for pCMSS33A (a chicken isolate), which exhibited a deletion in the region containing *dfrA14* (Figure 2).

## 3. Discussion

Non-typhoid salmonellosis is a major cause of foodborne illness worldwide. *Salmonella* contamination of poultry meat products occurs during slaughtering and processing, primarily due to exposure to bacteria-containing intestinal contents or improper handling [22]. In the present study, a high rate of *Salmonella* contamination was detected in poultry meat collected from retail markets across Korea. The prevalence of *Salmonella* spp. in retail chicken (79.0%) and duck meat (86.0%) was much higher than previously reported figures from Korea (21.2–42.3% in chicken meat and 51.3% in duck meat) [23,24,25,26]. Moreover, the proportion of *Salmonella* spp. in poultry meat was higher than that reported in China [1], Brazil [27], and the USA [28]. The higher frequency of *Salmonella* infections may be attributed to the isolation methods used in these studies. Most studies have isolated *Salmonella* spp. using xylose lysine deoxycholate (XLD) agar, a common isolation medium, whereas we used CHROMagar^TM^ *Salmonella* for isolation. Maddocks et al. [29] demonstrated the sensitivity and specificity of CHROMagar^TM^ Salmonella compared with other selective media. A clear difference was detected in the distribution of the predominant serotypes and STs of *Salmonella* between chicken and duck meat (Table 1). The predominant serovars and STs of *Salmonella* in chicken and duck meat were *S*. Infantis ST32 and *S*. Typhimurium ST19, respectively. *S*. Infantis recently became the most predominant serovar in broiler farms in Korea and is frequently identified as the causative agent of human salmonellosis worldwide [5,20]. In duck meat, *S*. Infantis ST32 was the third most frequently isolated serotype, with *S*. Brandenburg ST1954 and non-typable ST17, followed by non-typable ST33. Notably, *S*. Infantis duck isolates were detected only in duck meat from slaughterhouses that process both chickens and ducks. This suggests that cross-contamination of *S*. Infantis in duck meat may have occurred during the co-slaughtering process. Considering the serotype distribution in chicken and duck meat, *S*. Infantis isolated from duck meat may originate from chicken. This finding highlights the potential for *S*. Infantis transmission through duck meat and underscores the need for surveillance and proper management practices in poultry slaughterhouses.

The extensive use of antimicrobials in human and veterinary medicine for therapeutic or preventive purposes has promoted the emergence of AMR *Salmonella* strains that pose a threat to both animal and human health [30]. In the present study, we conducted an antimicrobial susceptibility test for the antimicrobials specified by the Korea Disease Control and Prevention Agency and the World Health Organization [31,32]. In results, high resistance rates were observed in poultry meat against NAL (68.0%), TET (54.9%), STR (54.5%), FIS (54.5%), and CHL (54.1%), with AMR profiles consistent with those of findings from previous studies [33,34,35]. Moreover, the resistance rates against nine antimicrobial agents, AMP, CTX, CHL, GEN, NAL, STR, FIS, TET, and SXT, were significantly higher in isolates from chicken meat than in those from duck meat (*p* < 0.001). A significantly higher proportion of MDR *Salmonella* was observed in chicken meat (79.7%) than in duck meat (18.6%). These results suggest that the selective pressure of antimicrobials may be higher in the chicken meat supply chain. The prevalence of MDR *Salmonella* is attributed to the overuse and misuse of antimicrobial agents for the prevention and treatment of diseases, which is consistent with a report that various antimicrobial agents are widely used in the poultry industry in Korea [36]. Moreover, 63.3% of the isolates from chickens and 8.1% of those from ducks were ESBL-producing *Salmonella* with MDR phenotypes. Consistent with the results reported by Kang et al. [19], the most prevalent MDR pattern among the ESBL-producing *S*. Infantis isolates was resistance to eight antimicrobial agents (Table 3). Despite the wide range of ESBLs, including TEM, SHV, CTX-M, and OXA β-lactamases, only *bla_CTX-M-65_* was found in *S*. Infantis ST32. ESBL-producing *S*. Infantis strains have been reported in several food isolates, raising concerns regarding the significant risk of zoonotic transmission to humans [14]. However, there is limited information on the genetic characteristics of ESBL-producing *S*. Infantis from poultry sources in Korea, particularly using recent advanced WGS techniques [19]. Therefore, WGS was conducted to better understand the genetic characteristics and relatedness of ESBL-producing *S*. Infantis isolates from retail poultry meat.

Phylogenetic analysis based on SNP alignment revealed genetic relatedness among some *S*. Infantis isolates irrespective of their geographical regions (Figure 1). Isolates from different provinces showed a significant clonal relationship, indicating the clonal spread of *S*. Infantis in retail poultry meat in Korea. Detection of AMR genes in ESBL-producing *S*. Infantis isolates revealed the presence of multiple resistance genes (Figure 1). The presence of AMR genes in the representative 28 isolates was not consistent with the phylogenetic clusters, indicating that the acquisition or loss of AMR genes adapted to the environment. Most of the AMR genes detected corresponded to the resistant phenotypes observed in this study. All isolates resistant to TET, NAL, and CTX harbored *tet(A)*, *gyrA* point mutations (D87Y), and *bla_CTX-M-65_*, respectively. In addition, 27 isolates were resistant to CHL and carried *floR*. Isolates carrying both trimethoprim (*dfrA14*) and sulfonamide (*sul1*) resistance genes were consistent with the SXT resistance phenotype. These findings demonstrate a clear correlation between the AMR genotype and phenotype. However, discrepancies were observed in aminoglycoside resistance, wherein the aminoglycoside genotype and phenotype did not match in some isolates. For instance, *aadA1* and *aac(3)-IVa* are known to confer resistance against STR and GEN, respectively [37]. However, consistent with Alzahrani et al. [33], false positive results for aminoglycoside resistance have been reported for susceptible isolates.

Several researchers have noted that the *S*. Infantis strain carries a megaplasmid, termed the “plasmid of emerging *S*. Infantis” (pESI), which contains multiple AMR, metal resistance, and virulence genes conferring significant adaptive advantages to the bacteria both within environments and hosts [38]. The pESI megaplasmid was first identified in Israel in 2008. Subsequently, numerous studies have shown the presence of pESI-carrying *S*. Infantis strains in food-producing animals, meats, and humans worldwide, raising concerns about the potential dissemination of MDR *S*. Infantis strains [14]. In Korea, based on the report of the Korean Veterinary Antimicrobial Resistance Monitoring System (KVARS), ESBL-producing MDR *S*. Infantis was first detected in *S*. Infantis isolates from slaughterhouses in 2020, and the detected isolates suddenly increased the following year. These ESBL-producing MDR *S*. Infantis strains were ST32 and typically carried *bla_CTX-M-65_*, which were strongly linked to ESI carrying pESI plasmid [19]. Soon after, *bla_CTX-M-65_* carrying a pESI-like plasmid in *S*. Infantis was reported in samples collected from egg packing facilities and broiler farms in 2022, through detection of *repA*, *ipf*, and K88-like genes using PCR [20,21]. However, in these studies, the clonal relatedness was analyzed using PFGE, which limits the ability to compare with strains reported by other research groups, as well as us. Moreover, some genetic components of pESI-like plasmids were screened by PCR. In the study by Kang et al. [19], ESBL-producing MDR *S*. Infantis isolated from slaughterhouses harbored *bla_CTX-M-65_* and IncFIB replicon, which is consistent with our findings. However, all ESI carrying a pESI plasmid isolated from egg packing facilities harbored two ESBL-producing genes (*bla_CTX-M-65_* and *bla_TEM-1_*), and their plasmid replicon type was IncP [21], which is a different type of pESI plasmid from those found in slaughterhouses [19] and meat in this study. These findings may indicate that ESI strains in broiler and layer farms carry different types of pESI-like plasmids in Korea. It is plausible that the previous study mistyped the plasmid replicon, as they used PCR-based replicon typing (PBRT) developed in 2005 [39]. The PBRT method was initially designed to detect the origin of replication (*oriV*) for IncP plasmids and the RepFIB replication protein A (*repB*) gene for IncFIB replicon-type plasmids. Several studies have demonstrated that pESI plasmids possess an *oriV* region of IncP-1α, which is typed as an IncP replicon when using the PBRT method [15,40]. Xu et al. [41] have demonstrated the sequence variability of the *repB* genes in the IncFIB replicon type, encompassing 7 primary types and 70 subtypes, which may account for the failure of targeting IncFIB in pESI-like plasmids with PBRT. Supporting this, the pESI-like plasmids detected in this study were found to possess IncFIB (pN55391), *repB*, and *oriV* (Figure 2, Supplementary Table S2), which is consistent with the findings of García-Soto et al. [42]. Based on the in silico PCR analysis, the replicon type of the pESI-like plasmids in this study was identified as IncP when applied with PBRT primers. Therefore, the PBRT method developed two decades ago should be replaced with a new method reflecting recent genetic information about *Salmonella*.

All 28 *S*. Infantis isolates analyzed in this study carried pESI-like plasmids harboring multiple resistance genes, including *bla_CTX-M-65_*. Previous studies have demonstrated that pESI-like megaplasmids commonly contain a diverse combination of AMR genes, particularly those conferring resistance to aminoglycosides, sulfonamides, tetracyclines, trimethoprim, and phenicols [14,16,21,37]. All 28 plasmids harbored yersiniabactin-encoding genes, which confer a siderophore-dependent iron uptake system that enhances *Salmonella* survival and virulence [38].

Globally, similar pESI-like megaplasmids have been reported in *S*. Infantis isolates from poultry and clinical samples in the United States, Israel, and several European countries. These plasmids commonly carry many genes for AMR, heavy metal resistance, oxidative stress, and virulence, in line with our findings [14,18,43]. The close genetic similarity (ANI > 99.9%) between Korean pESI-like plasmids and those from the United States suggests either a shared evolutionary origin or potential international transmission. As Alba et al. [44] noted, the structural variability of pESI plasmids likely reflects adaptation to environmental selective pressures. Recombination and rearrangement events may be evolutionarily favored, allowing plasmids to maintain core genetic determinants while acquiring additional resistance or virulence genes.

Notably, pESI-like plasmids are not exclusive to *S*. Infantis. Several studies have documented their horizontal transfer into non-Infantis *Salmonella* serotypes across different countries [45,46,47]. This highlights the potential for broader dissemination of MDR traits across serotypes throughout the poultry production chain.

Taken together, these results provide comprehensive WGS-based molecular characterization of *S*. Infantis in Korea, supporting the urgent need for ongoing surveillance across all sectors of poultry production. A nationally integrated monitoring system grounded in the One Health approach is essential to mitigate the risk of emerging MDR *Salmonella* lineages and to control their spread both within Korea and beyond its borders.

## 4. Materials and Methods

### 4.1. Sample Collection

In 2023, 300 poultry meat samples were collected from 20 retail markets across five provinces in Korea: Gyeonggi, Gyeongsang, Jeolla, Chungcheong, and Gangwon. Four retail markets were arbitrarily selected in each province, and ten chicken and five duck samples were collected from each retail market. Samples were stored in a cooled icebox at 4 °C and transported to the laboratory within 24 h of collection for isolation of *Salmonella* spp.

### 4.2. Isolation and Identification of Salmonella spp.

Each whole-body chicken or duck meat sample was rinsed in a sterile plastic bag containing 400 mL of buffered peptone water (BPW; BD, Sparks, MD, USA). Each rinsate was incubated at 37 °C for 24 h. Following that, 100 µL of the enriched BPW cultures were transferred into 10 mL of Rappaport–Vassiliadis (RV; BD) broth and incubated at 42 °C for 24 h [26]. A loopful of each RV culture was streaked onto CHROMagar^TM^ Salmonella (Paris, France) and incubated at 37 °C for 24 h. Up to three presumptive isolates were selected and subcultured on blood agar plates (Asan Pharm Co., Seoul, Korea) at 37 °C for 24 h. Identification of presumptive *Salmonella* isolates was determined by a TaqMan-based real-time PCR assay targeting the *invA* gene, as previously described [48].

### 4.3. Serotyping and Multi-Locus Sequence Typing

The serotypes of the *Salmonella* isolates were determined using multiplex PCR as described previously [49]. Multi-locus sequence typing (MLST) was conducted against seven housekeeping genes (*aroC*, *dnaN*, *hemD*, *hisD*, *purE*, *sucA*, and *thrA*), and STs were identified using the MLST database (https://pubmlst.org/organisms/salmonella-spp, accessed on 1 August 2023).

### 4.4. Antimicrobial Susceptibility Test

Antimicrobial susceptibility testing of *Salmonella* isolates against 16 antimicrobial agents was performed following the standardized protocols established by MFDS and the Animal and Plant Quarantine Agency [19,50,51]. The minimum inhibitory concentration (MIC) values were determined according to the broth microdilution method using the Sensititre^TM^ KRNV6F kit (Thermo Fisher Scientific, Waltham, MA, USA). The antimicrobial agents used were amoxicillin/clavulanic acid at a 2:1 ratio (AUG2), ampicillin (AMP), cefepime (FEP), cefotaxime (CTX), cefoxitin (FOX), ceftazidime (CAZ), chloramphenicol (CHL), ciprofloxacin (CIP), colistin (COL), gentamicin (GEN), meropenem (MERO), nalidixic acid (NAL), streptomycin (STR), sulfisoxazole (FIS), tetracycline (TET), and trimethoprim/sulfamethoxazole (SXT). *Escherichia coli* ATCC 25922 was used as the quality control reference strain in all tests. The MIC values of the isolates, except for STR, were interpreted according to the guidelines of the Clinical and Laboratory Standards Institute [52]. The breakpoint of STR was interpreted according to the National Antimicrobial Resistance Monitoring System [53]. Isolates resistant to CTX or CAZ or both were considered ESBL-producing *Salmonella* spp. for further identification as described below. When a strain was resistant to three or more antimicrobial classes, it was categorized as exhibiting MDR.

### 4.5. Characterization of the β-Lactamase-Encoding Genes

Detection of the β-lactamase-encoding genes was performed against the isolates resistant to CTX or CAZ or both. The presence of genes encoding different types of β-lactamases [Temoniera (TEM), sulphyldryl-variable (SHV), and cefotaxime (CTX-M)] was confirmed by PCR. Subtypes were determined by sequencing analysis as previously described [54,55]. For CTX-M-related genes, specific primer sets were used to identify each CTX-M group: CTX-M-1, CTX-M-2, CTX-M-8, CTX-M-9, and CTX-M-25. Sequence analyses were performed using BLAST at the National Center for Biotechnology Information (NCBI, https://blast.ncbi.nlm.nih.gov/Blast.cgi, accessed on 23 August 2023).

### 4.6. Whole-Genome Sequencing Analysis

A total of 28 representative ESBL-producing *S.* Infantis isolates were subjected to WGS to determine their genetic characteristics and phylogenetic relationships. WGS was performed by Macrogen (Seoul, Korea) following previously described [56].

To predict *Salmonella* serotype, in silico serotyping was performed using SeqSero2 (v.1.2.1) [57]. Single-nucleotide polymorphism (SNP) analysis was performed using the FDA-CFSAN SNP Pipeline. *S*. Infantis SINFA strain (GenBank accession number: N649235) was used as the reference genome for analysis [58]. A maximum likelihood (ML) tree was created using FastTree [59]. According to the framework for interpreting SNP analysis by the FDA-CFSAN, a cluster was defined when (1) there were 20 or fewer SNP differences, (2) the phylogenetic analysis showed a monophyletic relationship, and (3) there was a bootstrap support of 0.90 or higher [60].

Point mutations and genes leading to AMR were detected using AMRFinder Plus (version 3.11.26) [61]. PlasmidFinder (v. 2.1.6) was used to identify plasmid replicons [62]. Default parameters were used for all bioinformatics tools. The resulting data were visualized using iTOL (version 6.0; https://itol.embl.de/, accessed on 12 July 2024).

### 4.7. Detection and Reconstruction of pESI-like Plasmids

Plasmid sequences from the draft assemblies were typed and reconstructed using the MOB-Suite tool (v.3.1.8) with default parameters [63]. The reconstructed plasmids were annotated using Prokka (v.1.14.6). Plasmid MLST (pMLST) of the reconstructed plasmids was performed using pMLST (v.2.0) according to the IncI1 pMLST scheme on the Center for Genomic Epidemiology website (CGE, https://www.genomicepidemiology.org/, accessed on 13 August 2024). To determine the organization of AMR and virulence genes in the plasmids, AMRFinderPlus (v.3.11.26) and the Virulence Factor Database (VFDB; https://www.mgc.ac.cn/VFs/main.htm, accessed on 30 August 2024) were used, respectively [64]. Comparison and visualization of the characteristics of the plasmids were compared with those of the pESI-like plasmids available in the NCBI database using the OrthoANI algorithm [65] and visualized using the BLAST Ring Image Generator (BRIG, v.0.95) [66]. The WGS data of the pESI or pESI-like plasmids, pCVM44454 (GenBank accession number: CP016413.1), pN55391 (CP016411.1), 12037823-11_pESI-CTX-M-1 (OW849779.2), 119944_pESI (CP047882.1) and pS19 (CP092041.1), were used for the analysis.

### 4.8. Statistical Analysis

Chi-square tests were conducted to analyze significant differences in the isolation rates and AMR rates among *Salmonella* isolates from different samples using the SPSS program (v.26, Chicago, IL, USA). Statistical significance was set at a *p*-value of <0.05.

## 5. Conclusions

This study identified a high prevalence of *Salmonella* in retail poultry meat across Korea, with *S*. Infantis being the most dominant serotype in chicken meat. Notably, most *S*. Infantis isolates were ESBL-producing strains carrying *bla_CTX-M-65_* gene. WGS of 28 representative ESBL-producing *S*. Infantis isolates revealed close genetic relatedness among isolates from geographically distinct regions, suggesting clonal dissemination of these MDR strains throughout the poultry supply chain.

All 28 isolates harbored a pESI-like megaplasmid encoding multiple AMR, virulence, and stress resistance genes, which is consistent with global reports of emerging *S*. Infantis lineages. Importantly, our findings highlight previously underemphasized aspects, such as potential cross-contamination between chicken and duck meat during processing at shared slaughter facilities. This underscores the necessity of implementing stricter hygiene measures and targeted interventions in mixed processing environments.

Taken together, these results provide a comprehensive molecular characterization of *S.* infantis in Korea, underscoring the urgent need for continuous nationwide monitoring, expanded genomic surveillance, and improved biosecurity measures in poultry production. Future studies incorporating isolates from farms, slaughterhouses, and clinical sources are essential to build a more integrated One Health framework to tackle the dissemination of MDR *Salmonella* and mitigate risks to public health.

## Figures and Tables

**Table 1:** Prevalence, serotype, and sequence types (STs) of *Salmonella* in poultry meats.

Serotype	ST ^2^	Chicken Meat (%)	Duck Meat (%)	Total (%)
Infantis	ST32 *	106/200 (53.0)	7/100 (7.0)	113/300 (37.7)
Typhimurium	ST19 *	2/200 (1.0)	39/100 (39.0)	41/300 (13.7)
Enteritidis	ST11	7/200 (3.5)	0/100 (0.0)	7/300 (2.3)
Agona	ST13 *	21/200 (10.5)	1/100 (1.0)	22/300 (7.3)
Montevideo	ST4	4/200 (2.0)	0/100 (0.0)	4/300 (1.3)
Thompson	ST292 *	0/200 (0.0)	6/100 (6.0)	6/300 (2.0)
Stanley	ST321	0/200 (0.0)	1/100 (1.0)	1/300 (0.3)
Mbandaka	ST2133	3/200 (1.5)	0/100 (0.0)	3/300 (1.0)
Brandenburg	ST1954 *	0/200 (0.0)	7/100 (7.0)	7/300 (2.3)
Westhampton	ST14	3/200 (1.5)	0/100 (0.0)	3/300 (1.0)
Javiana	ST684 *	0/200 (0.0)	3/100 (3.0)	3/300 (1.0)
NT ^1^	ST17 *	0/200 (0.0)	7/100 (7.0)	7/300 (2.3)
	ST26	4/200 (2.0)	0/100 (0.0)	4/300 (1.3)
	ST33 *	0/200 (0.0)	13/100 (13.0)	13/300 (4.3)
	ST48	4/200 (2.0)	0/100 (0.0)	4/300 (1.3)
	ST203	3/200 (1.5)	0/100 (0.0)	3/300 (1.0)
	ST316	0/200 (0.0)	1/100 (1.0)	1/300 (0.3)
	ST543	1/200 (0.5)	1/100 (1.0)	2/300 (0.7)
Total		158/200 (79.0)	86/100 (86.0)	244/300 (81.3)

^1^ NT, non-typable. ^2^ ST, sequence type. * Significant differences were observed in the isolation rates between chicken and duck meat isolates (*p* < 0.05).

**Table 2:** Antimicrobial resistance (AMR) profiles of *Salmonella* in chicken and duck meats.

Antimicrobial Agents ^1^	MIC Break Point(μg/mL)	References ^2^	Origin of Isolates			
			Chicken Meat (n = 158)	Duck Meat (n = 86)	*p*-Value	Total (n = 244)
AMP	32	CLSI	103/158 (65.2) ^4^	14/86 (16.3)	<0.001	117/244 (48.0)
AUG2	32/16	CLSI	2/158 (1.3)	0/86 (0.0)	0.295	2/244 (0.8)
CAZ	16	CLSI	4/158 (2.5)	0/86 (0.0)	0.137	4/244 (1.6)
CHL	32	CLSI	121/158 (76.6)	11/86 (12.8)	<0.001	132/244 (54.1)
CIP	1	CLSI	2/158 (1.3)	3/86 (3.5)	0.242	5/244 (2.0)
COL	4	CLSI	1/158 (0.6)	1/86 (1.2)	- ^3^	2/244 (0.8)
CTX	4	CLSI	100/158 (63.3)	7/86 (8.1)	<0.001	107/244 (43.9) ^5^
FEP	16	CLSI	3/158 (1.9)	0/86 (0.0)	0.199	3/244 (1.2)
FIS	512	CLSI	120/158 (75.9)	13/86 (15.1)	<0.001	133/244 (54.5)
FOX	32	CLSI	6/158 (3.8)	0/86 (0.0)	0.067	6/244 (2.5)
GEN	16	CLSI	29/158 (18.4)	0/86 (0.0)	<0.001	29/244 (11.9)
MERO	4	CLSI	0/158 (0.0)	0/86 (0.0)	-	0/244 (0.0)
NAL	32	CLSI	122/158 (77.2)	44/86 (51.2)	<0.001	166/244 (68.0)
STR	32	NARMS	112/158 (75.9)	21/86 (24.4)	<0.001	133/244 (54.5)
SXT	4/76	CLSI	67/158 (42.4)	12/86 (14.0)	<0.001	79/244 (32.4)
TET	16	CLSI	121/158 (76.6)	13/86 (15.1)	<0.001	134/244 (54.9)
Multidrug resistance			126/158 (79.7)	16/86 (18.6)	<0.001	142/244 (58.2)

^1^ AMP, ampicillin; AUG2, amoxicillin/clavulanic acid 2:1 ratio; CAZ, ceftazidime; CHL, chloramphenicol; CIP, ciprofloxacin; COL, colistin; CTX, cefotaxime; FEP, cefepime; FIS, sulfisoxazole; FOX, cefoxitin; GEN, gentamicin; MERO, meropenem; NAL, nalidixic acid; STR, streptomycin; SXT, trimethoprim/sulfamethoxazole; TET, tetracycline. ^2^ CLSI, Clinical and Laboratory Standards Institution; NARMS, National Antimicrobial Resistance Monitoring System. ^3^ -, The *p*-value could not be calculated due to the identical values in both groups. ^4^ Data are presented as the number of resistant isolates/the number of tested isolates with percentages in parentheses. ^5^ All cefotaxime-resistant isolates were *S*. Infantis.

**Table 3:** AMR patterns of 107 extended-spectrum β-lactamase (ESBL)-producing *S*. Infantis.

AMR Phenotype *	Chicken Meat (%)	Duck Meat (%)	Total (%)
AMP-AUG2-CAZ-CHL-CTX-FEP-FIS-GEN-NAL-STR-SXT-TET	1 (1.0)	0 (0.0)	1 (0.9)
AMP-AUG2-CAZ-CHL-CTX-FEP-FIS-GEN-NAL-STR-TET	1 (1.0)	0 (0.0)	1 (0.9)
AMP-CAZ-CHL-CTX-FIS-GEN-NAL-STR-SXT-TET	1 (1.0)	0 (0.0)	1 (0.9)
AMP-CHL-CTX-FIS-FOX-GEN-NAL-STR-SXT-TET	2 (2.0)	0 (0.0)	2 (1.9)
AMP-CHL-CTX-FIS-FOX-NAL-STR-SXT-TET	1 (1.0)	0 (0.0)	1 (0.9)
AMP-CHL-CTX-FIS-GEN-STR-SXT-NAL-TET	17 (17.0)	0 (0.0)	17 (15.9)
AMP-CAZ-CHL-CTX-FIS-NAL-STR-TET	1 (1.0)	0 (0.0)	1 (0.9)
AMP-CHL-CTX-FIS-FOX-NAL-STR-TET	1 (1.0)	0 (0.0)	1 (0.9)
AMP-CHL-CTX-FIS-GEN-NAL-STR-TET	4 (4.0)	0 (0.0)	4 (3.7)
AMP-CHL-CTX-FIS-GEN-NAL-SXT-TET	2 (2.0)	0 (0.0)	2 (1.9)
AMP-CHL-CTX-FIS-STR-SXT-NAL-TET	37 (37.0)	7 (100.0)	44 (41.1)
AMP-CTX-FEP-FIS-FOX-NAL-STR-TET	1 (1.0)	0 (0.0)	1 (0.9)
AMP-CHL-CTX-FIS-NAL-STR-SXT	2 (2.0)	0 (0.0)	2 (1.9)
AMP-CHL-CTX-FIS-NAL-STR-TET	17 (17.0)	0 (0.0)	17 (15.9)
AMP-CHL-CTX-FIS-NAL-SXT-TET	1 (1.0)	0 (0.0)	1 (0.9)
AMP-CHL-CTX-NAL-STR-SXT-TET	1 (1.0)	0 (0.0)	1 (0.9)
AMP-CHL-CTX-GEN-NAL-TET	1 (1.0)	0 (0.0)	1 (0.9)
AMP-CHL-CTX-FIS-NAL-TET	2 (2.0)	0 (0.0)	2 (1.9)
AMP-CHL-CTX-FIS-NAL-STR	1 (1.0)	0 (0.0)	1 (0.9)
AMP-CHL-CTX-FOX-NAL	1 (1.0)	0 (0.0)	1 (0.9)
AMP-CHL-CTX-GEN-NAL	1 (1.0)	0 (0.0)	1 (0.9)
AMP-CHL-CTX-NAL-TET	4 (4.0)	0 (0.0)	4 (3.7)
Total	100/107 (93.5)	7/107 (6.5)	107/107

* AMP, ampicillin; AUG2, amoxicillin/clavulanic acid 2:1 ratio; CAZ, ceftazidime; CHL, chloramphenicol; CTX, cefotaxime; FEP, cefepime; FIS, sulfisoxazole; FOX, cefoxitin; GEN, gentamicin; NAL, nalidixic acid; STR, streptomycin; SXT, trimethoprim/sulfamethoxazole; TET, tetracycline.

## Data Availability

Genome sequence data of *S*. Infantis isolates have been deposited in the NCBI under BioProject accession number PRJNA1237260.

## Supplementary Materials

The following supporting information can be downloaded at: https://www.mdpi.com/article/10.3390/antibiotics14040366/s1, Table S1: Characteristics of *Salmonella* spp. isolated from retail poultry meats in this study; Table S2: Metadata of pESI-like plasmids in 28 ESBL-producing *S*. Infantis strains; Table S3: Pairwise ANI values for the pESI-like plasmids.
